# Successful 1:1 proportion ventilation with a unique device for independent lung ventilation using a double-lumen tube without complications in the supine and lateral decubitus positions. A pilot study

**DOI:** 10.1371/journal.pone.0184537

**Published:** 2017-09-14

**Authors:** Michał Kowalczyk, Sławomir Sawulski, Wojciech Dąbrowski, Luiza Grzycka-Kowalczyk, Edyta Kotlińska-Hasiec, Agnieszka Wrońska-Sewruk, Artur Florek, Rafał Rutyna

**Affiliations:** 1 1st Department of Anaesthesiology and Intensive Care, Medical University of Lublin, Lublin, Poland; 2 1st Department of Radiology and Nuclear Medicine, Medical University of Lublin, Lublin, Poland; ITALY

## Abstract

**Introduction:**

Adequate blood oxygenation and ventilation/perfusion matching should be main goal of anaesthetic and intensive care management. At present, one of the methods of improving gas exchange restricted by ventilation/perfusion mismatching is independent ventilation with two ventilators. Recently, however, a unique device has been developed, enabling ventilation of independent lungs in 1:1, 2:1, 3:1, and 5:1 proportions. The main goal of the study was to evaluate the device’s utility, precision and impact on pulmonary mechanics. Secondly- to measure the gas distribution in supine and lateral decubitus position.

**Materials and methods:**

69 patients who underwent elective thoracic surgery were eligible for the study. During general anaesthesia, after double lumen tube intubation, the aforementioned control system was placed between the anaesthetic machine and the patient. In the supine and lateral decubitus (left/right) positions, measurements of conventional and independent (1:1 proportion) ventilation were performed separately for each lung, including the following: tidal volume, peak pressure and dynamic compliance.

**Results:**

Our results show that conventional ventilation using Robertshaw tube in the supine position directs 47% of the tidal volume to the left lung and 53% to the right lung. Furthermore, in the left lateral position, 44% is directed to the dependent lung and 56% to the non-dependent lung. In the right lateral position, 49% is directed to the dependent lung and 51% to the non-dependent lung. The control system positively affected non-dependent and dependent lung ventilation by delivering equal tidal volumes into both lungs with no adverse effects, regardless of patient's position.

**Conclusions:**

We report that gas distribution is uneven during conventional ventilation using Robertshaw tube in the supine and lateral decubitus positions. However, this recently released control system enables precise and safe independent ventilation in the supine and the left and right lateral decubitus positions.

## Introduction

The maintenance of adequate blood oxygenation should be the main goal of each intra-operative anaesthetic management. This is achieved by maintaining adequate blood pressure and preventing ventilation/perfusion mismatch. Should these parameters not be met, oxygen delivery to the cells will be inadequate, leading to anaerobic metabolism and potential multi-organ failure. One of the reasons for clinical ventilation/perfusion mismatch can be pulmonary pathology, leading to so called shunt [1–3], another is—patient positioning on the operating table [4,5]. While young and healthy subjects can cope this scenario due to the efficiency of their physiological reflexes, in medicine, many surgical procedures involve elderly and critically ill patients with significant health impairments [6,7]. Additionally, the hypoxic vasoconstriction reflex is compromised by anaesthetic agents [8–10].

In the lateral decubitus position, greater perfusion occurs inside the dependent lung, while greater ventilation is found within the non-dependent one [1,2,11–13]. Data concerning tidal volume distribution in the lateral decubitus position are inconsistent and vary between 30% to 39% for the dependent lung. Presently, the only way to equalise uneven gas distribution is independent lung ventilation using two ventilators [1–3,12]. Recently, a unique device has been invented and patented by Polish engineers. This device enables dividing the inspired tidal volume into even volumes to both lungs during mechanical ventilation with double lumen oro-bronchial intubation (independent ventilation in a proportion of 1:1). This control system (called the 'tidal volume divider') can be easily attached to respirator or anaesthetic machine. Apart from allowing independent ventilation at a 1:1 ratio, it enables the division of the inspired volume in proportions of 2:1, 3:1 and 5:1 [14–16]. This capability provides a new perspective for treating intensive care unit patients with one-lung pathologies [3]. The second prospect for use is within general anaesthesia in the lateral decubitus position, especially in the surgical treatment of major concomitant diseases or involving the pathology of one lung. The main utility of the device could be prevention of volume shift between the lungs during lateral position ventilation.

The main hypothesis of the study was that the use of the device prevents the shift in tidal volume between the lungs during ventilation in the lateral decubitus position, as well as the evaluation of its impact on pulmonary mechanics. The second goal was to measure the distribution of inspired gases between the lungs in two positions, the supine and the lateral decubitus. Additionally, the impact of position changes on tidal volume distribution were assessed.

## Materials and methods

**Fig 1:**
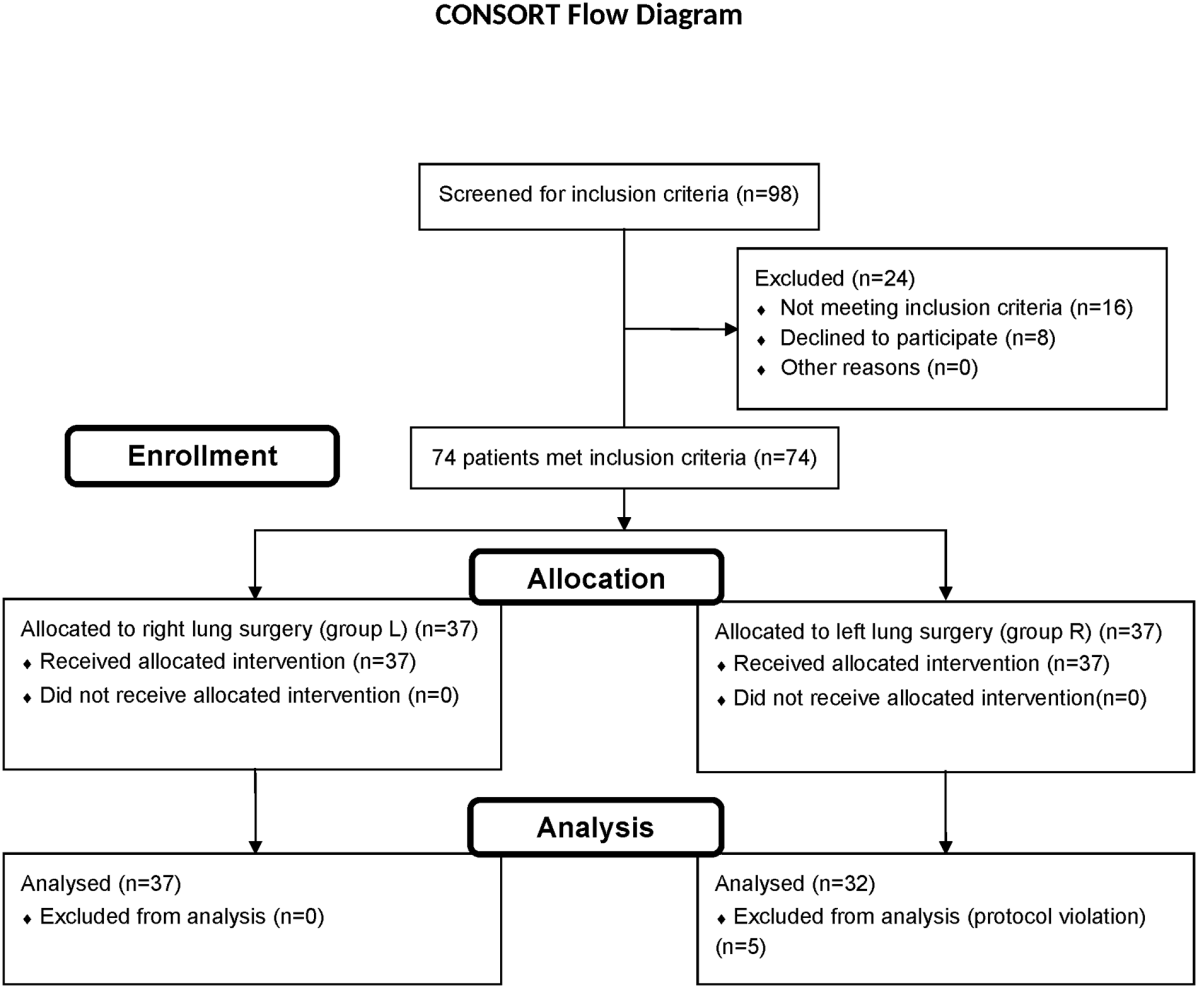
CONSORT flowchart.

Ethical approval for this study (Ethical Committee N° KE-0254/47/2009, approval date 26-02-2009) was provided by the Ethical Committee of Medical University of Lublin, Poland. Study protocol approved by Ethical Committee is attached as Supplementary Information. Study was registered as a trial at ClinicalTrials.gov, ID: NCT02786862. This trial was registered after patients requirement due to comply with WHO regulations. Reasons for delay in registration was that patients requirement has been done between March 2009 and February 2010 and at that time trial registration was not obligatory due to Polish regulations. The authors confirm that all ongoing and related trials for this intervention are registered. A flowchart of this study is presented in Fig 1.

After obtaining written consent, 69 ASA I and II patients who underwent elective thoracic surgery were eligible into the analysis. Inclusion criteria were as follow: elective thoracic surgery with double lumen tube intubation, assessment as ASA I or II, age above 18 years. Exclusion criteria were as follow: asthma or chronic obstructive pulmonary disease, history of thoracotomy, assessment as ASA III, difficult airway conditions, kyphoscoliosis or other alterations of chest wall or severe obesity. All patients were examined routinely 24 h before surgery. The typical tests were performed, as well as gasometric and spirometric exams for risk evaluation.

### Anaesthetic management

One hour before surgery, the patient-subjects received diazepam 0.15 mg kg^-1^ as premedication. After arriving at the operating theatre, standard monitoring system practices were applied, including heart rate (HR), systolic arterial pressure (SAP), diastolic arterial pressure (DAP), mean arterial pressure (MAP), and pulse oximetry (SpO_2_). Moreover, an intra-vein cannula was placed, and the infusion of multi-electrolytic fluid 5–10 ml kg^-1^ h^-1^ was started. After pre-oxygenation, atropine 0.5 mg and fentanyl 3 μg kg^-1^ were given, and the induction of anaesthesia with thiopentone 5–7 mg kg^-1^ was started. Suxamethonium was administered for neuromuscular blockade, and bronchial intubation with a Robertshow double lumen tube was performed. The left bronchus was intubated for right lung surgery, and the right bronchus was intubated for left lung surgery. Tube placement was checked via auscultation and fiberscope. Anaesthesia was maintained with sevoflurane, additional fentanyl doses were used if needed, and neuromuscular blockade was obtained with vecuronium 0.1 mg kg^-1^. Additionally, 0.1 mg kg^-1^ dose of morphine was given subcutaneously for postoperative pain control. Patients were also ventilated with O_2_ and AIR mixture, using the following settings: volume control intermittent positive pressure ventilation, FiO_2_ 0.4, Vt 6–10 ml/kg, and f 12–15 /min. Furthermore, end tidal CO_2_ was monitored due to normocapnia maintenance (4.0–5.3 kPa). At the end of surgery, intercostal blockade was brought about with 0.5% bupivacaine, with 5 ml for each nerve. Finally, the neuromuscular blockade was reversed with neostigmine 0.04 mg kg^-1^ and with atropine 0.01 mg kg^-1^.

### Ventilation measurements

**Fig 2:**
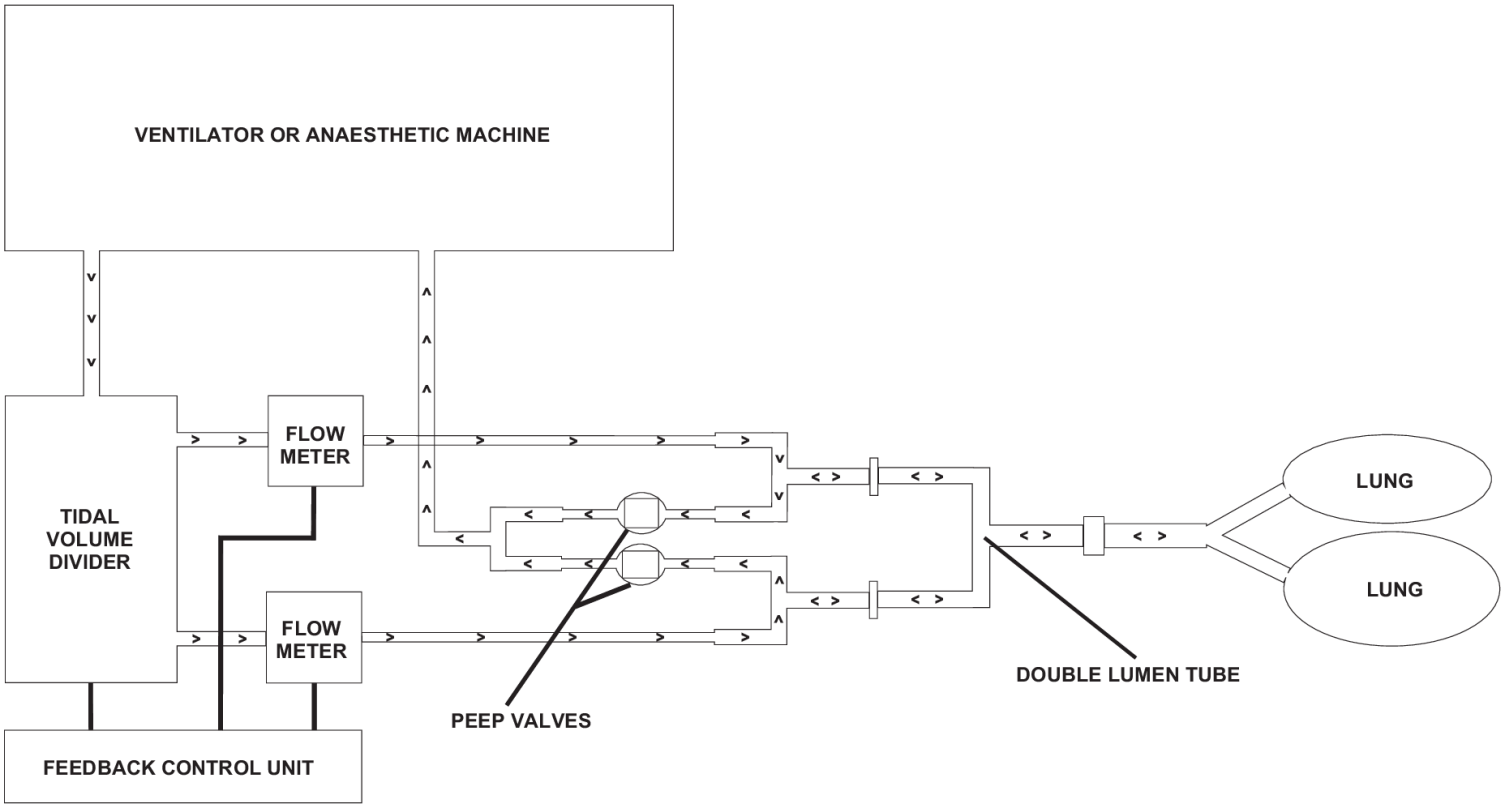
Scheme of the tidal volume divider.

After anaesthesia stabilisation, we used the unique control system, called the 'tidal volume divider'. This device was placed between the anaesthetic machine and the double lumen tube of the patient. This control system enables conventional ventilation (without any intervention from the control system, as the settings are defined on the ventilator), as well as independent ventilation with division of the tidal volume between the lungs in proportions of 1:1, 2:1, 3:1, and 5:1. It also enables selective positive end-expiratory pressure application ('PEEP') to each lung, using mechanical valves. With independent ventilation, settings such as frequency, tidal volume and inspiration time are defined by ventilator, and this system only controls the direction of tidal volume to each lung (as the control system is a flow divider) by using a differential pneumatic resistor. It also enables dependent and non-dependent lung ventilation in the lateral decubitus positions. Furthermore, the system monitors the expired volume, airway pressure and dynamic compliance of each lung, (Fig 2). This device was described and tested on mechanical lung models with a variety of lung model mechanics (compliance, resistance) and ventilation parameters (frequency, tidal volume) [15]. It was also tested clinically for its safety during our previous study [16]. The system was invented, developed and patented by a group of Polish engineers from the Nałęcz Institute of Biocybernetics and Biomedical Engineering, the Polish Academy of Sciences.

During the entire procedure, we constantly monitored the expired volume, the peak respiratory pressure and the dynamic compliance separately for each lung. These values were documented at each point of the study. We made measurements for conventional ventilation in the supine position; then, we began independent ventilation at a 1:1 proportion. Subsequently, we discontinued independent ventilation and moved the patient to the lateral decubitus position. In so doing, we divided our sample into two groups as follows: group L: patients moved to the left decubitus position due to right lung surgery, and group R: patients moved to the right decubitus position due to left lung surgery. We then made measurements for conventional anaesthetic practices and followed independent 1:1 proportion ventilation. Measurements at each point of study, covering also hemodynamic (MAP, HR) and oxygenation state (SpO_2_), were made after a 10 minute stabilisation period. Adverse effects of ventilation with control system were defined as follow: increase/decrease in blood pressure more than 20% of initial value, increase/decrease in heart rate more than 20% of initial value, pulse oximetry below 96%, peak inspiratory pressure more than 30 cm H_2_O. Subsequently, we disconnected the control system and performed typical anaesthetic procedures for thoracic surgery with a one-lung ventilation procedure. Any protocol violations resulted withdrawal from analysis (Fig 1). Study protocol (Polish and English versions) attached as S1 and S2 Files. Raw clinical data attached as S3 and S4 Files.

Statistical analysis was performed using STATISTICA software (StatSoft), with a significance level of 0.05. Sample size was calculated using STATISTICA Power Analysis module and determined at minimum level of 23 subjects per group. Assumptions for primary endpoint were as follow: tidal volume distributed to the dependent lung during conventional ventilation in the lateral decubitus position, on average (Mean 1) = 300 ± 30 ml (approximately 10% less than to the non-dependent lung). Tidal volume distributed to the dependent lung during independent 1:1 ventilation (with the device) in the lateral decubitus position, on average (Mean 2) = 330 ± 33 ml (approximately the same as to the non-dependent lung, without volume shift between the lungs), alpha = 0.05, power goal 0.9. Group characteristics values are presented as the mean and standard deviation due to normal distribution, and differences were tested using Student’s parametric t-test, with the exception of age, pO_2_ and sex. Age and pO_2_ differences were tested using the Mann-Whitney U-test due to non-parametric distribution. Sex differences are presented as numbers and percentages and were tested with the non-parametric chi-square test. Differences in MAP were tested using Student's parametric t-test due to normal distribution. Other values are presented as the median and range due to non-parametric distribution, and differences were tested using the non-parametric Wilcoxon test.

## Results

There were 69 ASA I and II patients enrolled in the analysis, with 32 in group R and 37 in group L. The thoracic procedures that the patients underwent were as follows: 22 (32%) cases involving partial lung resections, (due to: lung cancer—15 cases, carcinoid—4 cases, metastases—3 cases); 14 (20%) lobectomies, (due to: lung cancer 11 cases, bronchiectases—2 cases, lung absces—1 case); 13 (19%) explorative videothoracoscopies, (due to: tumour of unknown origin—6 cases, adenopathy—5 cases, metastasis spread—2 cases); 9 (13%) pneumonectomies, (due to lung cancer); 9 (13%) explorative thoracotomies, (due to: tumour of unknown origin—7 cases, metastases—2 cases); 1 (1.5%) bilobectomy, (due to lung cancer) and 1 (1.5%) mediastinal tumour resection, (due to mediastinal tumour).

There were no significant differences in patient characteristics (including spirometric and gasometric measurement results) between groups, as shown in Table 1.

**Table 1:** Patient characteristics.

	GROUP L	GROUP R	P value	TOTAL
No	37	32		69
Age (years)^a^	56.1 ± 9.9	56.9 ± 14.3	0.504	56.5 ± 12.0
Sex				
Women^b^	15 (40.5%)	8 (25.0%)	0.172	23 (33.3%)
Men^b^	22 (59.5%)	24 (75.0%)	0.172	46 (66.7%)
Weight (kg)^a^	74.6 ± 13.0	75.5 ±12.8	0.780	75.0 ± 12.8
Height (cm)^a^	168.4 ± 9.1	171.1 ± 8.8	0.217	169.7 ± 9.0
BMI (kg m^-2^)^a^*	26.2 ± 3.4	25.8 ± 4.0	0.633	26.0 ± 3.7
FVC (% as predicted)^a^†	101.0 ± 17.2	100.7 ± 17.2	0.934	100.8 ± 17.1
FEV_1_ (% as predicted)^a^‡	74.6 ± 5.4	75.7 ± 5.8	0.430	75.4 ± 5.6
PaO_2_ (mm Hg)^a^	77.9 ± 12.4	80.2 ± 13.9	1.000	78.6 ± 12.6
PaCO_2_ (mm Hg)^a^	38.5 ± 3.5	35.9 ± 5.3	0.126	37.6 ± 4.1

^a^ Values are presented as the mean ± standard deviation;

^b^ Values are presented as numbers and, in parentheses, as percentages.

*BMI; Body Mass Index

^†^FVC; forced vital capacity

^‡^FEV_1_; forced expiratory volume in 1 second

### Tidal volume distribution

Our results showed that during conventional ventilation using Robertshow tube in the supine position, the right lung received a larger volume of air in comparison to the left: 53±6% vs. 47±6% (p = 0.000), respectively (as % of total tidal volume). There was no peak pressure difference between the lungs. The dynamic compliance differed, with the right lung being more compliant (on average). Independent ventilation at a proportion of 1:1 ensured the equal division of tidal volume to each lung (50±1% each), without significant changes in peak pressure and dynamic compliance (Table 2).

**Table 2:** Biomechanical parameters during conventional and independent (1:1) ventilation in the supine position.

	CONVENTIONAL VENTILATION			INDEPENDENT VENTILATION IN 1:1 PROPORTIONS		
	SUPINE POSITION (ALL, n = 69)					
PARAMETER	LEFT LUNG MED (IQR)	RIGHT LUNG MED (IQR)	p value	LEFT LUNG MED (IQR)	RIGHT LUNG MED (IQR)	p value
Volume (ml)	290 (65)	330 (75)	<0.001	300 (50)	305 (55)	0.138
Peak pressure (cm H_2_O)	16.0 (4.0)	16.0 (4.0)	0.219	16.0 (5.0)	16.0 (5.0)	0.996
Dynamic compliance (ml cm H_2_O^-1^)	22.4 (8.0)	25.0 (8.0)	0.013	24.0 (8.3)	24.0 (8.5)	0.537

MED, median; IQR, interquartile range

After the change in position, patients in group L were placed into the left decubitus position. These test subjects shown an uneven tidal volume distribution, with 44±6% to the left (lower, dependent) and 56±2% to the right (upper, non-dependent) lung (p = 0.000), without peak pressure differences between lungs but with higher non-dependent lung compliance. However, independent ventilation at a proportion of 1:1 brought about an equal division of tidal volume to each lung (50±2% each), without significant changes in peak pressure and dynamic compliance (Table 3).

**Table 3:** Biomechanical parameters during conventional and independent (1:1) ventilation in the left and right decubitus positions.

	CONVENTIONAL VENTILATION			INDEPENDENT VENTILATION IN 1:1 PROPORTIONS		
	**LEFT DECUBITUS POSITION (GROUP L, n = 37)**					
**PARAMETER**	**LEFT LUNG** **MED (IQR)**	**RIGHT LUNG** **MED (IQR)**	**p value**	**LEFT LUNG** **MED (IQR)**	**RIGHT LUNG** **MED (IQR)**	**p value**
Volume (ml)	270 (70)	340 (50)	<0.001	300 (40)	310 (45)	0.086
Peak pressure (cm H_2_O)	17.0 (2.0)	17.0 (3.0)	0.075	17.0 (3.0)	16.0 (3.0)	0.364
Dynamic compliance (ml cm H_2_O^-1^)	20.0 (7.4)	26.8 (8.0)	<0.001	20.5 (6.0)	25.0 (8.3)	0.111
	**RIGHT DECUBITUS POSITION (GROUP R, n = 32)**					
Volume (ml)	315 (85)	300 (60)	0.035	300 (73)	320 (70)	0.104
Peak pressure (cm H_2_O)	15.5 (5.0)	16.0 (4.5)	0.625	14.5 (5)	15.0 (4.5)	0.466
Dynamic compliance (ml cm H_2_O^-1^)	23.9 (8.4)	22.5 (8.6)	0.221	23.3 (10.5)	23.9 (8.0)	0.483

MED, median; IQR, interquartile range

The right decubitus reposition also induced an unequal tidal volume distribution, with 51±5% to the left (upper, non-dependent) and 49±5% to the right (lower, dependent) (p = 0.035). There were no peak pressure and dynamic compliance differences between these patients' lungs. Independent ventilation in a proportion of 1:1 distributed 50±1% of the tidal volume to the each lung, without any peak pressure and dynamic compliance changes (Table 3).

### Impact of position change

Position change impact analysis for the tidal volume distribution also revealed that the left and right decubitus positions induce unequal gas distribution. Accordingly, the left position distributed 44±6% of the tidal volume distribution to the dependent lung and 56±6% to the non-dependent lung, while the right position directed 49±5% of this distribution to the dependent lung and 51±5% to the non-dependent one. There were no significant peak pressure changes according to the position change, but dynamic compliance decreased in the dependent lungs and increased in the non-dependent lungs in both positions (Table 4).

**Table 4:** Impact of position change on biomechanical parameters.

LEFT LUNG										RIGHT LUNG								
**SUPINE vs. LEFT DECUBITUS POSITION (GROUP L, n = 37)**																		
**DEPENDENT LUNG**									**NON-DEPENDENT LUNG**									
Supine	Left decubitus		Supine	Left decubitus		Supine	Left decubitus		Supine		Left decubitus		Supine	Left decubitus		Supine	Left decubitus	
Volume (ml) [% VT]	Volume (ml) [% VT]	p	P_PEAK_ (cm H_2_O)	P_PEAK_ (cm H_2_O)	p	C_DYN_ (ml cm H_2_O^-1^)	C_DYN_ (ml cm H_2_O^-1^)	p	Volume (ml) [% VT]		Volume (ml) [% VT]	p	P_PEAK_ (cm H_2_O)	P_PEAK_ (cm H_2_O)	p	C_DYN_ (ml cm H_2_O^-1^)	C_DYN_ (ml cm H_2_O^-1^)	p
300 (60) [49%]	270 (70) [44%]	<0.001	16.0 (4.0)	17.0 (2.0)	0.352	21.4 (9.0)	20.0 (7.4)	0.001	300 (80) [51%]		340 (50) [56%]	<0.001	16.0 (3.0)	17.0 (3.0)	0.754	24.0 (8.3)	26.8 (8.0)	0.021
**SUPINE vs. RIGHT DECUBITUS POSITION (GROUP R, n = 32)**																		
**NON-DEPENDENT LUNG**									**DEPENDENT LUNG**									
Supine	Right decubitus		Supine	Right decubitus		Supine	Right decubitus		Supine		Right decubitus		Supine	Right decubitus		Supine	Right decubitus	
Volume (ml) [% VT]	Volume (ml) [% VT]	p	P_PEAK_ (cm H_2_O)	P_PEAK_ (cm H_2_O)	p	C_DYN_ (ml cm H_2_O^-1^)	C_DYN_ (ml cm H_2_O^-1^)	p	Volume (ml) [% VT]		Volume (ml) [% VT]	p	P_PEAK_ (cm H_2_O)	P_PEAK_ (cm H_2_O)	p	C_DYN_ (ml cm H_2_O^-1^)	C_DYN_ (ml cm H_2_O^-1^)	p
283 (70) [46%]	315 (85) [51%]	<0.001	15.0 (5.5)	15.5 (5.0)	0.218	23.4 (8.3)	23.9 (8.4)	0.042	338 (73) [54%]		300 (60) [49%]	<0.001	15.0 (5.0)	16.0 (4.5)	0.088	26.3 (7.8)	22.5 (8.6)	0.002

Values are presented as medians and in parentheses as interquartile ranges, additionally in square brackets volumes are presented as percent of total tidal volume

% VT, percent of total tidal volume; P_PEAK_, peak pressure; C_DYN_, dynamic compliance

### Impact of independent ventilation

Independent ventilation in a proportion of 1:1 in the supine position using Robertshaw tube increased the left lung compliance after ventilation, but there were no significant changes in the right lung. There was no effect of independent ventilation institution on hemodynamic parameters, such as mean arterial pressure, heart rate and oxygenation state, as measured by pulse oximetry in the supine position. However, independent ventilation in the left decubitus position increased the left (dependent) lung compliance and decreased that of the right (non-dependent) lung. Similar to the results observed in the supine position, in the left decubitus position, independent ventilation had no significant influence on hemodynamic and oximetry measurements. In the right decubitus position, increased right (dependent) lung compliance occurred without any other significant changes (Table 5). As above, there were no changes in the hemodynamic or oximetry parameters after initiation of independent ventilation.

**Table 5:** Changes in biomechanical parameters after independent (1:1) ventilation.

LEFT LUNG										RIGHT LUNG								
**SUPINE POSITION (ALL, n = 69)**																		
CONV	1:1		CONV	1:1		CONV	1:1		CONV		1:1		CONV	1:1		CONV	1:1	
Volume (ml) [% VT]	Volume (ml) [% VT]	p	P_PEAK_ (cm H_2_O)	P_PEAK_ (cm H_2_O)	p	C_DYN_ (ml cm H_2_O^-1^)	C_DYN_ (ml cm H_2_O^-1^)	p	Volume (ml) [% VT]		Volume (ml) [% VT]	p	P_PEAK_ (cm H_2_O)	P_PEAK_ (cm H_2_O)	p	C_DYN_ (ml cm H_2_O^-1^)	C_DYN_ (ml cm H_2_O^-1^)	p
290 (65) [47%]	300 (50) [50%]	<0.001	16.0 (4.0)	16.0 (5.0)	0.393	22.4 (8.0)	24.0 (8.3)	<0.001	330 (75) [53%]		305 (55) [50%]	<0.001	16.0 (4.0)	16.0 (5.0)	0.909	25.0 (8.0)	24.0 (8.5)	0.893
**LEFT DECUBITUS POSITION (GROUP L, n = 37)**																		
270 (70) [44%]	300 (40) [50%]	<0.001	17.0 (2.0)	17.0 (3.0)	0.721	20 (7.4)	20.5 (6.0)	<0.001	340 (50) [56%]		310 (45) [50%]	<0.001	17.0 (3.0)	16.0 (3.0)	0.902	26.8 (8.0)	25.0 (8.3)	<0.001
**RIGHT DECUBITUS POSITION (GROUP R, n = 32)**																		
315 (85) [51%]	300 (73) [50%]	0.014	15.5 (5.0)	14.5 (5.0)	0.616	23.9 (8.4)	23.3 (10.5)	0.158	300 (60) [49%]		320 (70) [50%]	0.018	16.0 (4.5)	15.0 (4.5)	0.949	22.5 (8.6)	23.9 (8.0)	0.001

Values are presented as medians and in parentheses as interquartile ranges, additionally in square brackets volumes are presented as percent of total tidal volume

CONV, conventional ventilation; 1:1, independent ventilation in 1:1 proportions; % VT, percent of total tidal volume; P_PEAK_, peak pressure; C_DYN_, dynamic compliance

There were no registered adverse effects during the whole study protocol.

## Discussion

Our data with respect to gas distribution in the supine position were consistent with established literature, as Baehrendtz and Klingstedt [12] showed the same findings (53% to the right and 47% to the left) and so did Bindslev et al. [13] (52% to the right and 48% to the left). These studies were also performed on anaesthetised human subjects with double-lumen intubation. Baehrendtz et al. present slightly different values in two studies, with 55% vs. 45% in the first [1] and 54 vs. 46% in the second [2], but these findings involved intensive care unit patients with acute bilateral lung disease.

The findings of inspired gas distribution in anaesthetised subjects in the lateral decubitus position were less consistent. Presently, only a few reports assessed inspired gas distribution during conventional ventilation with anaesthetised subjects in the lateral decubitus position, and these were mostly without left/right side distinction. In contrast, our study revealed differences in gas distribution between the left and right decubitus positions. It must be noted that Bindslev et al. [13] only made their measurements in the left lateral position. Their results showed a 61% gas distribution to the non-dependent lung and 39% to the dependent lung. In two of the studies made by Baehrendtz et al. [1,2] involving intensive care patients, the distribution was 70% to the non-dependent lung and 30% to the dependent lung. One of these studies did not distinguished between left or lateral position [1], and the second one only assessed the left lateral position [2]. All of the studies previously mentioned were made on small sample populations, between 7 to 11, while our population sample was for 69 patients, with higher statistical value.

Differences in gas distribution between lungs in the supine position probably do not have a great clinical relevance, but are informative and could supplement literature findings, while uneven gas distribution in the lateral decubitus position during general anaesthesia with artificial ventilation lowers the functional residual capacity (FRC) [17, 18] causing alveolar collapse [19,20] and compression atelectasis [4,20]. The second issue is the perfusion inequality caused by gravitational force [11,21] and what is more dynamic hyperinflation diverts perfusion towards the dependent hypo-inflated lung [22,23]. The above mentioned factors can increase ventilation/perfusion mismatch and venous admixture [1,2,12] leading to a decrease in oxygen tension and increase the risk for organ failure. Additionally, anaesthetic agents can impair hypoxic pulmonary vasoconstriction reflex [7–10]. One of the ways to improve ventilation/perfusion inequality is the initiation of independent ventilation, which has been previously shown to improve ventilation/perfusion matching and decrease venous admixture [1–3,12].

The ideal situation is to divert the tidal volume in proportion to regional perfusion. The independent ventilation of each lung with two synchronised ventilators meets this need. As shown by Baehrendtz et al. [12] on anaesthetised patients, independent ventilation with equal tidal volume decreases shunt by 26% and increases arterial oxygen tension by 27%.

We should mention that general PEEP application can improve gas exchange, but it decreases cardiac output [24] and diverts flow into the less compliant lung [25]. Only selective PEEP into the dependent lung can improve ventilation/perfusion matching [26].

The application of independent ventilation with two ventilators is very difficult, requiring specialised equipment and additional staff. Currently, we have a new opportunity to institute independent ventilation with tidal volume equalisation by using the unique device designed, developed and patented by Polish engineers. The use of this device is practical, comfortable and safe, as demonstrated during our current study, the maximal pressure did not cross the safe level of 30 cmH_2_O. Additionally, the device enables selective PEEP application [27]. Moreover, the use of independent ventilation did not cause any adverse hemodynamic effects, as we showed in both the supine and the left and right lateral decubitus positions. Furthermore, oxygenation measured by pulse oximetry did not decrease with independent ventilation, and while we hypothesised that oxygen tension should improve, we were limited in arterial oxygenation and degree of shunt measurements.

We believe that particular benefits should be obtained during thoracic surgery procedures requiring lateral decubitus positioning, especially in long lasting operations when alveoli collapse could occur [28]. Position concerned ventilation/perfusion mismatch could be fully controlled with this system. Furthermore, in addition to 1:1 proportioning for patient ventilation, this device allows 2:1, 3:1 and 5:1 proportioning. We feel that this could be clinically useful in patients with greater impairment. Hypoxemia occurs during one lung ventilation in the lateral position for thoracic surgery in 5–10% patients [29] and could be exaggerated among chronically ill subjects. For example, independent ventilation with 5:1 proportioning in the lateral decubitus position for thoracic surgery could enable the performance of surgical procedures without hypoxemia. This outcome requires non-dependent lung ventilation with some interference due to surgery but should be possible. This issue needs further study. Another application could involve intensive care patients with unilateral lung pathology and with great compliance differences between lungs. Sawulski et al. [30] described the case of young trauma patient with unilateral lung pathology who had been successfully treated through 1:1 and 2:1 proportion ventilation applications with this device. Improved gas exchange enabled collapsed lung expansion without traumatic repercussions on the compliant lung and likely saved the patient from surgical lobectomy.

A very interesting clinical application could be expected in patients after single-lung transplantation, as Pilcher et al. [3] reported, treatment with independent lung ventilation after one lung transplantation leads to a satisfactory long-term outcome.

The limitation of this study was lack of detailed hemodynamic and oxygen status measurements due to low efficiency of non-invasive methods and the higher risk for patients while using the invasive one. The second limitation was that all subjects had conventional ventilation prior to independent 1:1 ventilation, what potentially could cause carry-over effect to independent 1:1 ventilation. We assumed that conventional ventilation should not affect patients lungs in a lasting manner, so when we changed ventilation to independent in 1:1 proportions, all biomechanical lungs properties should be the same. Of course these assumptions apply to lungs without any serious pathologies as in our study: only patient ASA I and II were included. What is more, to minimize bias risk 10 minutes stabilisation period at each point of study was applied before measurements. Another issue is lack of randomization and the two groups R and L could not be well balanced in patient-subject characteristics by the study design. We assumed that it might be impossible to make randomization meeting ethical committee approval. For example, if we would assign right lung surgery patient to group R we should move patient to the right decubitus position, then take measurements and move to left decubitus position for surgery proceeding. Additional changes in position carry unnecessary risk for patient and should be avoided. To minimize bias caused by lack of baseline groups characteristics adjustment we enrolled only ASA I and II patients with all exclusions. As these assumptions were correct there should not be any baseline differences between groups, what was confirmed by our statistical analysis (Table 1). As we used two types of Robertshow tubes: left and right, it could potentially bias our measurements due to inhaled gas distribution. Other disadvantage of studied device is that during ventilation we can use only one ventilator mode while during independent ventilation with two respirators two different modes can be applied, separately to each lung.

## Conclusions

Concluding, in our study we revealed uneven gas distribution during conventional ventilation using Robertshow tube in general anaesthetised humans in the supine, as well as in lateral decubitus positioning. Of these, the most disadvantageous gas distribution was in the left lateral decubitus position. The control system, designed, developed and patented by Polish engineers, enabled precise, safe and simple independent (in 1:1 proportions) ventilation in the supine, as well as in the left and right lateral decubitus positions without any serious adverse effects.

## Supporting information

S1 File Study protocol Polish version. (DOCX)

S2 File Study protocol English version. (DOCX)

S3 File Raw clinical data—main data. (STA)

S4 File Raw clinical data—spirometry and general data. (STA)

S5 File Consort checklist. (DOC)
