# Supplementary material for: Successful 1:1 proportion ventilation with a unique device for independent lung ventilation using a double-lumen tube without complications in the supine and lateral decubitus positions. A pilot study
Source: PLoS One. 2017 Sep 14;12(9):e0184537. doi: 10.1371/journal.pone.0184537 (PMC5598983; doi:10.1371/journal.pone.0184537)
Supplement: S1 File — (DOCX) [file pone.0184537.s001.docx]

**PROTOKÓŁ BADANIA:**

**Kryteria włączenia:** wiek powyżej 18 lat, ASA I lub II, planowy zabieg torakochirurgiczny

**Kryteria wykluczenia:** astma, przewlekła obturacyjna choroba płuc, torakotomia w wywiadzie, ASA III, trudne drogi oddechowe, kyfoskolioza lub inne zniekształcenia klatki piersiowej, duża otyłość (BMI > 35).

**POSTĘPOWANIE ANESTEZJOLOGICZNE**

**24 godz. przed zabiegiem:** rutynowe badanie, rutynowe badania laboratoryjne, gazometria i spirometria.

**Premedykacja:** jedna godzina przed zabiegiem: 0.15 mg kg^-1^.

**Monitorowanie w trakcie procedury:** częstość tętna (HR), skurczowe ciśnienie tętnicze (SAP), rozkurczowe ciśnienie tętnicze (DIAP), średnie ciśnienie tętnicze (MAP), pulsoksymetria (SpO_2_), końcowowydechowy CO_2_.

**Przed indukcją znieczulenia:** kaniula dożylna, wlew płynu wieloelektrolitowego 5-10 ml kg^-1^ h^-1^, preoksygenacja.

**Indukcja znieczulenia:** atropina 0.5 mg, fentanyl 3 µg kg^-1^, tiopental 5-7 mg kg^-1^, suksametonium 1 mg kg^-1^, intubacja rurką dwuświatłową Robertshowa - do lewego oskrzela dla operacji prawego płuca, do prawego oskrzela dla operacji lewego płuca. Kontrola przez osłuchiwanie i fiberoskopowo.

**Podtrzymanie znieczulenia:** sewoflurane i fentanyl (w zależności od potrzeb), wekuronium 0.1 mg kg^-1^.

**Wentylacja:** mieszaniną O_2_/POWIETRZE, ustawienia: wentylacja objętościowo zmienna przerywanym dodatnim ciśnieniem, FiO_2_ 0.4, Vt 6-10 ml/kg, f 12-15 /min.

**Na końcu zabiegu:** morfina w dawce 0.1 mg kg^-1^, blokada międzyżebrowa z 0.5% bupiwakainy 5 ml na każdy nerw, neostygmina 0.04 mg kg^-1^ z atropiną 0.01 mg kg^-1^.

**Pomiary wentylacji:**

**Po ustabilizowaniu znieczulenia:** umieść urządzenie do rozdziału objętości oddechowej między aparatem do znieczulenia a rurką dwuświatłową.

Wykonaj wszystkie pomiary zgodnie z kartą pomiarów umieszczoną na następnej stronie.

**Następnie:** odłącz urządzenie do rozdziału objętości oddechowej i przeprowadź typowe znieczulenie do zabiegu torakochirurgiczego z wentylacją jednego płuca.

**Na końcu:** wykonaj kopię karty anestezjologicznej rutynowo używanej w szpitalu.

**KARTA POMIARÓW:**

Imię i Nazwisko: Wiek (lata): Data:

Waga (kg): Wzrost (cm):

UWAGI:

| **SPIROMETRIA** | |
| --- | --- |
| FVC (% należnej) | FEV_1_ (% należnej) |
|  |  |

FVC - natężona pojemność życiowa

FEV_1_- natężona objętość wydechowa 1 sekundowa

| **GAZOMETRIA** | | |
| --- | --- | --- |
| pH | pO2 (mmHg) | pCO2 (mmHg) |
|  |  |  |

OPERACJA: **LEWEGO PŁUCA / PRAWEGO PŁUCA***

Rodzaj zabiegu torakochirurgicznego:

**POZYCJA NA PLECACH:**

|  | P max  (cm H_2_O) | P mean  (cm H_2_O) | PEEP  (cm H_2_O) | dyn comp  (ml/cm H_2_O) | V (ml) | MAP (mmHg) | HR/min. | SpO_2_ (%) |
| --- | --- | --- | --- | --- | --- | --- | --- | --- |
| **WENTYLACJA SWOBODNA** | | | | | | | | |
| płuco L |  |  |  |  |  |  |  |  |
| płuco P |  |  |  |  |  |  |  |  |
| **WENTYLACJA WYMUSZONA W STOSUNKU 1:1** | | | | | | | | |
| płuco L |  |  |  |  |  |  |  |  |
| płuco P |  |  |  |  |  |  |  |  |
| **POZYCJA NA BOKU: PRAWYM* / LEWYM*** | | | | | | | | |
| **WENTYLACJA SWOBODNA** | | | | | | | | |
| płuco L |  |  |  |  |  |  |  |  |
| płuco P |  |  |  |  |  |  |  |  |
| **WENTYLACJA WYMUSZONA W STOSUNKU 1:1** | | | | | | | | |
| płuco L |  |  |  |  |  |  |  |  |
| płuco P |  |  |  |  |  |  |  |  |
| **WENTYLACJA WYMUSZONA W STOSUNKU 2:1** | | | | | | | | |
| płuco L |  |  |  |  |  |  |  |  |
| płuco P |  |  |  |  |  |  |  |  |
| **WENTYLACJA WYMUSZONA W STOSUNKU 3:1** | | | | | | | | |
| płuco L |  |  |  |  |  |  |  |  |
| płuco P |  |  |  |  |  |  |  |  |
| **WENTYLACJA WYMUSZONA W STOSUNKU 5:1** | | | | | | | | |
| płuco L |  |  |  |  |  |  |  |  |
| płuco P |  |  |  |  |  |  |  |  |

P - ciśnienie, PEEP - dodatnie ciśnienie końcowowydechowe, dyn comp - podatność dynamiczna, V - objętość oddechowa, MAP - średnie ciśnienie tetnicze, HR - częstość akcji serca, SpO_2_ - pulsoksymetria, L - lewe, P - prawe

* - zaznacz właściwe
