# Supplementary material for: Successful 1:1 proportion ventilation with a unique device for independent lung ventilation using a double-lumen tube without complications in the supine and lateral decubitus positions. A pilot study
Source: PLoS One. 2017 Sep 14;12(9):e0184537. doi: 10.1371/journal.pone.0184537 (PMC5598983; doi:10.1371/journal.pone.0184537)
Supplement: S2 File — (DOCX) [file pone.0184537.s002.docx]

**STUDY PROTOCOL:**

**Including criteria:** age above 18 years old, ASA I or II assessment, elective thoracic surgery.

**Excluding criteria:** asthma, chronic obstructive pulmonary disease, history of thoracotomy, assessed as ASA III, with difficult airway conditions, kyphoscoliosis or other alterations of chest wall, severe obesity (BMI > 35).

**ANAESTHETIC MANAGEMENT**:

**24 h before surgery:** routine examination, routine laboratory tests, gasometric and spirometric exams

**Premedication:** one hour before surgery: diazepam 0.15 mg kg^-1^

**Monitoring during procedure:** heart rate (HR), systolic arterial pressure (SAP), diastolic arterial pressure (DAP), mean arterial pressure (MAP), pulse oximetry (SpO_2_), end tidal CO_2_

**Before induction of anesthesia:** intra-vein cannula, the infusion of multi-electrolytic fluid 5-10 ml kg^-1^ h^-1^, pre-oxygenation.

**Anesthesia induction:** atropine 0.5 mg, fentanyl 3 µg kg^-1^, thiopentone 5-7 mg kg^-1^, suxamethonium 1 mg kg^-1^, intubation with a Robertshow double lumen tube - to left bronchus for right lung surgery, to right bronchus for left lung surgery. Checking via auscultation and fiberscope.

**Anaesthesia maintenance:** sevoflurane and fentanyl (as much as need), vecuronium 0.1 mg kg^-1^.

**Ventilation:** O_2_/AIR mixture, settings: volume control intermittent positive pressure ventilation, FiO_2_ 0.4, Vt 6-10 ml/kg, f 12-15 /min.

**At the end of surgery**: 0.1 mg kg^-1^ dose of morphine, intercostal blockade with 0.5% bupivacaine 5 ml for each nerve, neostigmine 0.04 mg kg^-1^ with atropine 0.01 mg kg^-1^.

**Ventilation measurements:**

**After anaesthesia stabilisation:** place the tidal volume divider between the anaesthetic machine and the double lumen tube of the patient.

Make all measurements due to measurement chart given on the next page.

**Subsequently**: disconnect the tidal volume divider and perform typical anesthetic procedure for thoracic surgery with a one-lung ventilation.

**At the end:** Please make copy of the anesthetic chart routinely used at hospital.

**MEASUREMENT CHART:**

Name and Surname: Age (years): Date:

Weight (kg): Height (cm):

REMARKS:

| **SPIROMETRIC TEST** | |
| --- | --- |
| FVC (% as predicted) | FEV_1_ (% as predicted) |
|  |  |

FVC - forced vital capacity, FEV_1_- forced expiratory volume in 1 second

| **GASOMETRIC TEST** | | |
| --- | --- | --- |
| pH | pO2 (mmHg) | pCO2 (mmHg) |
|  |  |  |

SURGERY: **LEFT LUNG / RIGHT LUNG***

Type of the thoracic procedure:

**SUPINE POSITION:**

|  | P max  (cm H_2_O) | P mean  (cm H_2_O) | PEEP  (cm H_2_O) | dyn comp  (ml/cm H_2_O) | V (ml) | MAP (mmHg) | HR/min. | SpO_2_ (%) |
| --- | --- | --- | --- | --- | --- | --- | --- | --- |
| **CONVENTIONAL VENTILATION** | | | | | | | | |
| lung L |  |  |  |  |  |  |  |  |
| lung R |  |  |  |  |  |  |  |  |
| **INDEPENDENT AT 1:1 PROPORTION VENTILATION** | | | | | | | | |
| lung L |  |  |  |  |  |  |  |  |
| lung R |  |  |  |  |  |  |  |  |
| **DECUBITUS POSITION: RIGHT* / LEFT*** | | | | | | | | |
| **CONVENTIONAL VENTILATION** | | | | | | | | |
| lung L |  |  |  |  |  |  |  |  |
| lung R |  |  |  |  |  |  |  |  |
| **INDEPENDENT AT 1:1 PROPORTION VENTILATION** | | | | | | | | |
| lung L |  |  |  |  |  |  |  |  |
| lung R |  |  |  |  |  |  |  |  |
| **INDEPENDENT AT 2:1 PROPORTION VENTILATION** | | | | | | | | |
| lung L |  |  |  |  |  |  |  |  |
| lung R |  |  |  |  |  |  |  |  |
| **INDEPENDENT AT 3:1 PROPORTION VENTILATION** | | | | | | | | |
| lung L |  |  |  |  |  |  |  |  |
| lung R |  |  |  |  |  |  |  |  |
| **INDEPENDENT AT 5:1 PROPORTION VENTILATION** | | | | | | | | |
| lung L |  |  |  |  |  |  |  |  |
| lung R |  |  |  |  |  |  |  |  |

P - pressure, PEEP - positive end expiratory pressure, dyn comp - dynamic compliance, V - tidal volume, MAP - mean arterial pressure, HR - heart rate, SpO_2_ - pulse oximetry, L - left, R - right

* - mark as appropriate
